# Biosorption of Cr (III) from Polluted Water Using *Pennisetum clandestinum* Hochst (Kikuyo)

**DOI:** 10.3390/molecules30030682

**Published:** 2025-02-04

**Authors:** Amanda Rosa Maldonado-Farfán, Uriel Raul Fernández-Bernaola, Rocio Victory Vargas-Robles, Jessica Gabriela Villasante-Muñoz, Martin Daniel Trejo-Valdez

**Affiliations:** 1 Departament of Chemical Engineering, Universidad Nacional de San Antonio Abad del Cusco, Cusco 08003, Peru; amanda.maldonado@unsaac.edu.pe (A.R.M.-F.); 110122@unsaac.edu.pe (R.V.V.-R.); 170792@unsaac.edu.pe (J.G.V.-M.); 2 Instituto Politecnico Nacional, Gustavo A. Madero 07700, Mexico; martin.trejo@laposte.net

**Keywords:** kikuyu, biosorption, Cr (III), fur effluents, kinetics

## Abstract

Given the abundance of kikuyu biomass resulting from the pruning of green areas, the aim of this study was to evaluate its use as a biosorbent (BK) for Cr (III) removal from polluted waters. The biomass was activated using H_2_SO_4_ (1.25%) and NaOH (3.25%). The characterization methods were Fourier-Transform Infrared Spectroscopy (FTIR), scanning electron microscopy/energy-dispersive spectroscopy (SEM/EDS), and Brunauer–Emmett–Teller (BET) analysis. Our results confirmed the presence of active groups on BK, such as –OH, -C=C-, -C=O, and -C-O-, with an increase of 1308.58% in specific surface area, as well as the presence of chromium on the biosorbent after adsorption process. The adsorption capacity (q) was tested in a jar test as a function of biomass granulometry, dose (BK), and the pH of the solution; the best response was 47.9 mg/g at a pH of 5.5, a biosorbent dose of 0.5 g/L, and a biosorbent size of 100 μm. The effect of pH was positive; by increasing the pH, the adsorption capacity increased. However, the effect of the biosorbent dose and size was negative, as when increasing the dose and granulometry, the adsorption capacity decreased. In addition, the kinetic process was studied, where the removal data were better fitted for the pseudo-second-order kinetic model, confirming that the adsorption mechanism was chemisorption. The adsorption capacity was 37.6 mg/g for industrial wastewater. The possibility of using kikuyu within the circular economy was demonstrated and suggests its application in continuous systems for real-world environmental conditions.

## 1. Introduction

Caring for the environment is both a necessity and a requirement for sustainable development, a responsibility that each society assumes for future generations. Metallurgical, electroplating, tanning, leatherworking, pulp production, battery, and paper printing ink industries, among others, are of high environmental significance due to the presence of Cr (III) and Cr (VI) in their effluents. These effluents, ranging from tens to hundreds of mg/L, cause negative environmental impacts [1,2,3,4]. The tanning industry has been extensively developed in the south of Peru. Leatherworking is another important form of artisanal production in the Cusco Region, generating effluents with a pollution load of Cr (III) equivalent to one-third of the chromium salt used [5]. These effluents can be treated via conventional treatment processes such as chemical precipitation, flotation, ion exchange, membrane separation, electrocoagulation, and adsorption [6,7]. Among these methods, biosorption is a physicochemical phenomenon where solute particles, in solution, accumulate themselves on the surface of biomass (alive or dead) [7,8,9].

The amount of waste that we generate globally and the negative effects that it has make it necessary to change the production model and adopt a model based on the circular economy. This model is inspired by nature, and it could be said that the circular economy is based on the fact that the waste that we generate must serve as raw material to produce new products. Producing goods under this model has important benefits, such as reducing the exploitation of natural resources and emissions. Studies aimed at reusing this waste in a sustainable manner have made significant progress on a laboratory scale and are beginning to be implemented on an industrial scale. Therefore, increasing the reuse of agricultural waste can effectively reduce pollution. Peru has a wide range of plant species that are interesting to study as biosorbents for removing contaminants in water. There have been suggestions to reorient efforts toward species of plants that are typically considered weeds, grow during the rainy season, lack potential for medicinal applications, or are not registered as a traditional medicine from their country of origin. Kikuyu (*Pennisetum clandestinium* Hochst) belongs to these types of biomasses. Belonging to the Poaceae family and originating from Africa, it has been reported in Peru, where it is considered an invader of natural forests, crops, agricultural and coastal areas, and roadsides, and in some cases, it is used in dairy production systems [10]. Biomasses contain chemical functional groups in their structures that retain pollutants through electrical attractions, van der Waals forces, or their chemical nature. Approximately 60–90% of biomass is composed of polymeric substances that could form stable complexes with heavy metals through their functional groups; for example, carboxylic acids, phosphates, sulfates, amines, and amides [11,12,13,14,15,16,17]. It is interesting to note that the properties and characteristics of surface biomass depend on, among other aspects, the prior treatment of the natural material, which could increase its adsorption performance [18]. Thus, acid and/or basic treatment of biomaterials enables an increase in their adsorption capacity, as it leads to a rupture of their polymer chains and the emergence of a greater number of functional groups from cellulose [19]. Without those treatments, natural biomass could not obtain better properties for sorption; for example, higher porosities and more functional group availabilities. However, one disadvantage of this technique is related to the use of toxic reagents, such as sulfuric acid or sodium hydroxide. It is recommended to replace those reagents with more environmentally friendly reagents or use them at lower concentrations.

Therefore, in this study, we aimed to evaluate the biosorption capacity of Cr (III) from polluted waters using the residual biomass of kikuyu (*Pennisetum clandestinum* Hochst) activated via acid and basic treatment. For this reason, we conducted a physical, chemical, and morphological characterization of the BK biosorbent, evaluating the influence of the biosorbent particle size, dose, and pH to determine the best conditions of Cr (III) adsorption. In addition, we established a mathematical model to relate the variables under study, as well as a kinetic model from the saturated biosorbent with Cr (III), treated under various pH conditions. Finally, the Cr (III) adsorption capacity of the effluent obtained from the ASAPES fur industry was evaluated using the biosorbent.

## 2. Results and Discussion

### 2.1. Biosorbent Characterization

**Figure 1:**
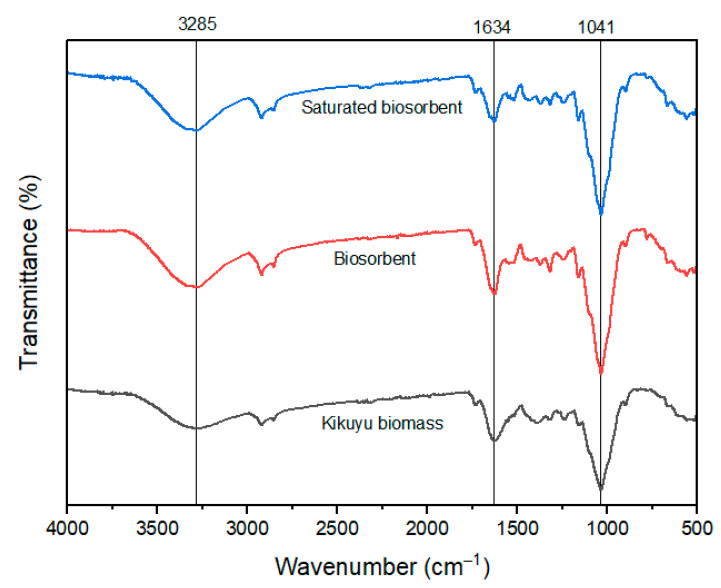
FTIR spectra. Kikuyu biomass (B); biosorbent (BK), and saturated biosorbent (BKS).

Figure 1 shows the FTIR spectra of the B, BK, and BKS, respectively, where a large number of absorption peaks are observed that characterize the presence of the functional groups, -OH (3285 cm^−1^), -C=O (1634 cm^−1^), -C-O- (1041 cm^−1^), and C=C- (1690 cm^−1^), confirming their lignocellulosic nature [20,21,22]. The first three functional groups, where the oxygen atom is present with its two pairs of free electrons, provide the BK points of high electron density, which become active adsorption centers. In the case of group C=C-, the electron density arises from the π bond; therefore, group C=O has a higher electronic density. The difference between the spectra occurs with lower values of transmittance percentage (higher absorbance percentage), indicating a better arrangement of the negatively charged functional groups in the BK with respect to the B. This is attributed to acid–base treatment [23] and its screening with Cr (III) cations (BKS), whereby the active attraction of these cations towards the surface of the BK in the adsorption process occurs. This is due to complexation with functional groups that contain oxygen, such as the hydroxyl group, and electrostatic attraction using carbonyl, confirming effective adsorption of a chemical nature [24].

**Figure 2:**
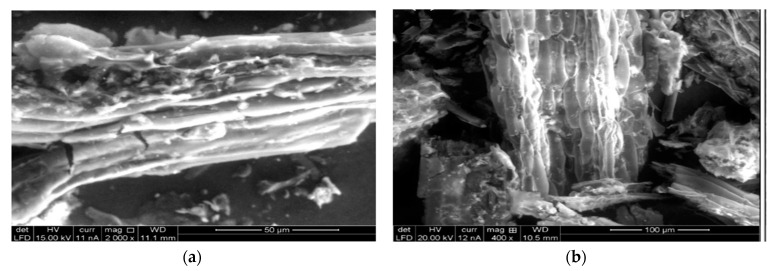
SEM images. (**a**) Biosorbent (BK); (**b**) saturated biosorbent (BKS).

Through BET analysis, it was possible to estimate that 1 g of kikuyu biomass has 0.0303 m^2^ of specific surface area, increasing to 0.4268 m^2^ (an increase of 1308.58%) due to the acid treatment solubilizing the hemicellulose and the basic treatment rupturing its polymer chains and then increasing the porosity and surface area of the biosorbent, thus increasing the quantity of ionic functional groups [20,21,22,23,24,25]. These attributes are typical of biosorbents; for instance, higher specific surface areas for the biomasses of banana piths, pineapple stems, and bamboo stems were determined [26]; these results could be attributed to the chemical modifications that those biomasses underwent. The morphological micrograph (a) of the BK (Figure 2) shows an elongated, striated, and laminated solid structure formed by horizontal rough plates with the presence of randomly distributed surface undulations and cavities, providing surface texture and different levels of microporosity, which, thus, enhances adsorption capacity [21,23]. The micrograph of the BKS (b), however, presents an ordered honeycomb-shaped structure, which could be due to the saturation of the pores with Cr (III). Similar structures are shown with the yellow passion fruit shell bioadsorbent [27].

EDS elemental analysis of the BKS (Table 1) confirmed the presence of Cr (III), aligning with its organic nature, which is due to the high presence of carbon.

The results of the characterization using FTIR spectroscopy, the BET method, and scanning electron microscopy (SEM/EDS) show the potential of the biosorbent to remove Cr (III) from polluted waters.

### 2.2. Biosorption Tests

**Figure 3:**
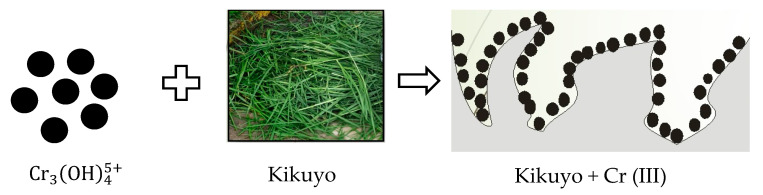
Cr (III) biosorption process.

Figure 3 shows the biosorption process of chromium. At pH between 4.8 and 5.5, there are ${Cr}_{3}{\left(OH\right)}_{4}^{5+}$ species principally [28]. Those ions Cr (III) interact with functional groups from kikuyu.

Table 2 shows the average results of the adsorption capacity (q).

An analysis of the results shows that a minimum Cr (III) adsorption capacity was achieved in test 6, corresponding to 21.3 ± 3.6 mg/g under the lower pH conditions (4.8) of the polluted solution, a higher dose (1 g/L), and higher biosorbent size (212 μm). The highest adsorption capacity (47.9 ± 4.5 mg/g) was achieved in test 10, which included the highest pH (5.5), a lower biosorbent dose (0.5 g/L), and a lower biosorbent size (106 μm). This performance could be due to the fact that at a higher pH, there is less competition between hydrogen ions and Cr (III) cations [27]. Similarly, if the particle size is smaller, the active groups are more available for the absorbate and facilitate diffusion into the pores. However, a lower dose involves the presence of the active sites necessary for the adsorption of Cr (III), with a lower presence of unsaturated active sites. Higher doses of the biosorbent could cause agglomeration or superposition on the active sites, thus preventing the desired electrostatic interactions that would affect the decrease in adsorption capacity [29]. Table 3 shows the comparison of the adsorption capacity of kikuyu and other biosorbents.

The results obtained in this research (47.9 mg/g) exceed the performance of a Cr (III) biosorbent prepared with native microalgae consortium (4.44 mg/g); *Algae spirogyra* spp. (30.21 mg/g); chitosan microfiber bayberry tannin (20.90 mg/g); *Zea mays* seed chaff (14.68 mg/g); and aerobic granules (37.8 mg/g) [21,30,31,34,36] under two different treatments. Higher Cr (III) adsorption capacities were tested using yellow passion fruit peel; *Cymbopogon flexuosus*; and Arribadas algae and rose waste biomass petals as biosorbents [27,32,33,35].

The authors demonstrated the selectivity of a biosorbent obtained from *Pennisetum clandestinium* for Pb (II) ions (139.35 mg/g) [7]. Their result may be due to the relationship between the average pore size of the biosorbent and the radius of the ion.

### 2.3. Statistical Analysis

Table 4 shows the estimated effects of the independent variables on the adsorption capacity (q).

In Table 4, it can be observed that the effect of pH is positive; thus, by increasing the pH from 4.8 to 5.5, the adsorption capacity increases by 3.86 mg/g on average. However, the effect of the biosorbent dose is negative, as increasing the dose from 0.5 to 1 mg/L results in an average decrease in adsorption capacity of 17.74 mg/g. Similarly, the effect of the biosorbent size is negative, as increasing the size from 106 to 212 μm results in an average decrease in adsorption capacity of 3.99 mg/g. Regarding the interactions, the effects of the variables are negative in the case of pH–dose (AB) and pH–biosorbent size (AC) interactions but positive in the case of dose–size (BC) interactions, where the adsorption capacity increases on average by 1.77 mg/g.

**Figure 4:**
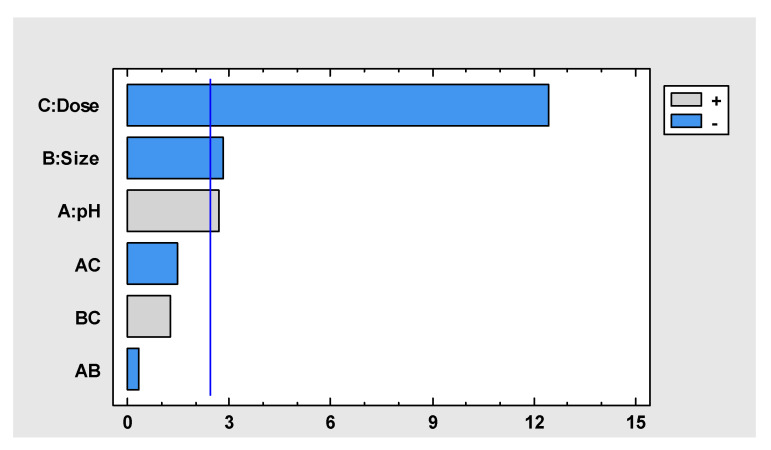
Standardized Pareto diagram for q.

Figure 4 shows a standardized Pareto diagram for the adsorption capacity. In the graph, it can be inferred that the three variables (biosorbent dose, size, and pH) exhibit significant effects, because they crossed the blue line, with respect to the adsorption capacity.

The pH of the solution has an important role in the adsorption process, as it determines the surface charge, degree of ionization, and adsorption process [29]. Thus, in several studies, higher adsorption capacities were reported with a pH in the range of 4.5 to 5.0 [23,27,28,30,31,32,35,36], despite the competition between H+ and Cr (III) ions for active sites on the biosorbent. Additionally, the difference between adsorption capacities obtained from other studies reported with this work is probably because higher biosorbent doses were tested; for example, 8 g/L of yellow passion fruit shell; 70 g/L of *Cymbopogon flexuosus* immobilized in alginate sodium; 20 g/L of eggshell; 5 g/L of *Algae spirogyra* spp.; 10 g/L of Arribadas algae; and 1 g/L of rose waste biomass [27,30,32,33,35,37]. At pH values higher than 7, hydrolysis could take place, leading to a decrease in adsorption capacity [27].

The mathematical model that relates the variables under study, with R^2^ = 85.34%, is as follows:

(1) q=−14.1694+16.6548·pH+15.9119·D−0.0196429·T−12.0476·pH·D−0.0132525·pH·T+0.0669811·D·T

This model explains 85.34% of the experimental uncertainty, demonstrating the goodness of fit.

### 2.4. Kinetics Study

The data from the kinetic study show that the adsorption capacity q increases with time, with a rapid increase observed at the beginning and within the first minutes, and then equilibrium is reached in less than 80 min (q = 41.42 mg/g).

**Figure 5:**
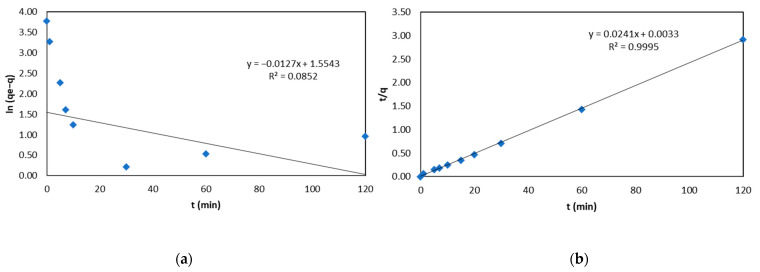
Data fitted to kinetic models: (**a**) pseudo first order (**b**) pseudo second order.

The pseudo-first-order kinetics model did not fit the experimental data, as shown in Figure 5a, considering a determination coefficient of 8.52%. The pseudo-second-order kinetics model best fits the experimental data (k_2_ = 0.194 g/mg min), as shown in Figure 5b, explaining 99.95% of the uncertainty. These results indicate that the biosorption process depends on the metal ion and the biosorbent [27], providing evidence that the predominant adsorption mechanism is chemisorption, which involves the link formation between functional biosorbent groups and chromium ions (adsorbate) in a monolayer. In addition, it is based on the influence of valence forces through the sharing or exchange of electrons between Cr (III) ions and the available sites on the biosorbent surface [29], which also indicates that the limiting stage is the adsorption itself, rather than mass transfer [38].

Table 5 shows the kinetic constant models. For example, so many studies investigated yellow passion fruit shells, pineapple plant stems, chitosan microfiber bayberries, rose waste biomass, and aerobic granules as biosorbents that also reported pseudo-second-order kinetics as a biosorption mechanism. In comparison, the values of the pseudo-second-order rate constants (k) of these studies (7.52 × 10^−3^; 0.014; 2 × 10^−3^; 0.017 and 3.3 × 10^−3^ g/mg∙min, respectively) were lower than those of the biosorbent (BK) under study [27,31,35,36,39]. Thus, the adsorption velocity of Cr (III) on kikuyu is higher.

### 2.5. Bioadsorption Tests with Fur Industry Effluent

In the biosorption tests with samples from the ASAPES fur industry, with a 0.5 mg/L dose of biosorbent, a pH of 5.5, and a biosorbent size of 106 μm, an adsorption capacity of 37.6 ± 0.2 mg/g was achieved, corresponding to 78.5% of the adsorption capacity with synthetic water (47.9 mg/g). This result could be attributed to the possible interferences caused by suspended solids, salts, fats, and other compounds typical of the fur industry, as well as the presence of other ions that compete for the active sites of the biosorbent. In addition, in an investigation into a native microalgae consortium used to remove Cr (III) from two different sources, real tannery wastewater and synthetic water, it was possible to establish that the Cr (III) adsorption capacity of real wastewater is 91.22% of the adsorption capacity with synthetic water, which is attributed to the selectivity of the Cr (III) ion by the biosorbent [21].

## 3. Materials and Methods

### 3.1. Biosorbent Preparation

A total of 1 kg of kikuyu (*Pennisetum clandestinum* Hochst) was collected from the gardens at the Universidad Nacional de San Antonio Abad del Cusco (UNSAAC), washed with deionized water, dried in a KYNTEL forced convection oven at 80 °C for 24 h, weighed (EUROTECH precision analytical digital scale, model FSF-A2204B, repeatability ± 0.1 mg), and ground. The obtained biomass (B) was activated using soxhlet equipment via acid treatment with H_2_SO_4_ (p.a. 97%, SCHARLAU brand) at 1.25% for 60 min and then dried [7,26]. This was followed by basic treatment with NaOH (98.8% SCHARLAU brand) at 3.25% for 60 min and subsequent drying. Then, the activated biomass was sifted through certified ASTM standard Tyler meshes Nos. 70, 100, and 140 and stored in a desiccator. The resulting product constitutes the biosorbent (BK).

### 3.2. Characterization Methods

The active functional groups of the B, BK, and saturated biosorbent with Cr (III) (BKS) were identified using Fourier-Transform Infrared Spectroscopy (FTIR) in the range of 380 cm^−1^ to 4000 cm^−1^, employing a PERKIN ELMER spectrophotometer (Waltham, MA, USA) with Spectrum 10 software Origin version 2024b [21].

The measurement of the specific surface areas and characteristics of the mesopores of the B and BK were carried out using Brunauer–Emmett–Teller (BET) analysis in a MICROMERITICS (Norcross, GA, USA) surface area analyzer. Prior to the N_2_ sorption experimental runs, each sample was degassed with He through a heat treatment at 200 °C for 6 h under a vacuum.

Using scanning electron microscopy (SEM) [6], the morphology and the presence of characteristic elements were elucidated for the BK and BKS sample spectra through photomicrographs. For this analysis, a scanning electron microscope was used (SEM/EDS, FEI QUANTA 650, detector: EDAX model OCTANE PRO (Hillsboro, OR, USA)).

### 3.3. Biosorption Tests with Synthetic Polluted Water

A standard stock solution with a concentration of 250 ppm Cr (III) was prepared from Cl_3_Cr∙6H_2_O salt (99%, Merck (Rahway, NJ, USA)). The biosorption tests were carried out using jar test equipment (DAIHAN SCIENTIFIC, Model JT-M6C (Seoul, Republic of Korea)), employing synthetic polluted water containing 50 mg/L Cr (III) (a concentration that exceeds the concentrations of chromium from fur effluents in the region) with a volume of 300 mL. The stirring speed was 150 rpm, with tests conducted for 2 h. We utilized Multivariable Factorial Experimental Design 2^3^. Table 6 shows the independent variables and their respective levels. The total number of trials was 8, plus 3 midpoints. Realizing 2 replicates for each treatment, in total, 33 experiments were tested.

At the end of each test, the samples were filtered using Wattman N°40 filter paper, where the residual Cr (III) content was analyzed via a Flame Atomic Absorption Spectrophotometer (THERMO SCIENTIFIC, AA ICE 3300) at 357.87 nm. The number of chromium ions adsorbed (q) in mg Cr/g biosorbent during the series of batch experiments was determined using the mass balance equation [9]

(2) $$q=\frac{V}{m}\left(C_{i}-C_{f}\right)$$

where q is the adsorption capacity of Cr (III) in mg/g, C_f_ is the final concentration of Cr (III) in ppm, C_i_ is the initial concentration of Cr (III) in ppm, V is the volume of the solution in liters (L), and m is the mass of biosorbent in grams (g). The biosorbent size (T), dose (D), and pH conditions that reached the highest q were fixed as parameters for the following tests.

### 3.4. Statistical Analysis

The data obtained were statistically treated with Statgraphics Centurion 18 software to determine the effect of the independent variables (T, D, and pH) on q, the mathematical prediction model, and its goodness of fit.

### 3.5. Kinetic Study

The biosorption kinetics of Cr (III) on BK were analyzed by fitting the experimental data to the pseudo-first-order and pseudo-second-order kinetic models [22]. For this purpose, 300 mL of a polluted solution with Cr (III) (50 mg/L) was prepared, with the biosorbent dose (D) and pH determined in the previous tests, and stirred at a speed of 150 rpm. At different times, 10 mL aliquots were taken up to 2 h and then filtered, and the concentration of Cr (III) was subsequently determined via atomic absorption. Two replicates were performed for each test. The linearized forms of the kinetic models are expressed using Equations (3) and (4)

(3) $$ln\left(q_{e}-q_{t}\right)=lnq_{e}-k_{1}t$$

(4) $$\frac{t}{q_{t}}=\frac{1}{k_{2}{q_{e}}^{2}}+\frac{t}{q_{e}}$$

where q is the adsorption capacity at time t (mg/g), t is the contact time (min), q_e_ is the adsorption capacity at equilibrium (mg/g), k_1_ is the constant rate (min^−1^), and k_2_ is the pseudo-second-order rate constant (g∙mg^−1^∙min^−1^).

### 3.6. Biosorption of Fur Industry Effluent

Finally, the Cr (III) adsorption process using the BK was tested in a real sample. This sample was collected from the ASAPES fur industry (Sicuani-Cusco-Peru), specifically being an effluent from the tanning bath process. A composite sample of 3 L was taken and filtrated using Wattman No. 40 paper. Then, 300 mL of water was treated at a stirring speed of 150 rpm for a duration of 2 h. The residual concentration of Cr (III) was measured via AA, and the adsorption capacity q was calculated using Equation (2).

## 4. Conclusions

In conclusion, it was demonstrated that activated residual kikuyu biomass with acid and basic hydrolysis produced a higher superficial area and exposed more functional groups that were effective for removing Cr (III) from polluted waters. FTIR and SEM/EDS analyses identified the presence of Cr (III) in the biosorbent. In the adsorption process, the three variables under study (biosorbent dose, size, and pH) exhibited a significant influence on the adsorption capacity at a pH of 5.5, a biosorbent dose of 0.5 g/L, and a biosorbent size of 100 μm. Maximum adsorption capacities Y_máx_ of 47.9 mg/g and 37.6 mg/g were achieved for synthetic water and industry effluents, respectively. The biosorption data were better fitted for the pseudo-second-order kinetic model, confirming that the adsorption mechanism was chemisorption (K_2_ = 0.194 g∙mg^−1^∙min^−1^). The results of our study indicate that residual biomass could be utilized for the removal of Cr (III) from tannery effluents, thereby reducing the cost of effluent treatments. Future research should be aimed at the application of the biosorbent in continuous systems and its subsequent scaling up at the pilot plant level in real-world environmental conditions.

## Figures and Tables

**Table 1:** Elemental analysis (EDS) of the saturated biosorbent (BKS).

Element	Unit	Zone No. 1	Zone No.2	Zone No. 3		
		Area 1	Area 1	Area 1	Area 2	Area 3
Carbon, C	%	54.11	55.88	67.85	68.27	63.73
Oxygen, O	%	42.17	41.27	30.83	30.47	33.47
Chromium, Cr	%	0.48	1.02	0.65	0.65	0.86
Aluminum, Al	%	0.41	-	-	0.23	-
Silicon, Si	%	2.82	0.49	0.4	0.39	1.45
Calcium, Ca	%	-	0.66	0.27	-	0.49

**Table 2:** Adsorption capacity (mg/g).

Nº	pH	Dose (g/L)	Size (μm)	Cf (mg/L)	q (mg/g)
1	5.15	0.75	150	28.3 ± 2.23	28.9 ± 3
2	5.5	0.5	212	29.7 ± 1.21	40.6 ± 2.4
3	4.8	0.5	212	31.9 ± 1.89	36.1 ± 3.8
4	5.5	1	106	24.8 ± 3.15	25.2 ± 3.2
5	4.8	1	106	26.0 ± 0.53	24.0 ± 0.5
6	4.8	1	212	28.7 ± 3.62	21.3 ± 3.6
7	5.5	1	212	26.5 ± 3.86	23.5 ± 3.9
8	4.8	0.5	106	29.8 ± 2.95	40.4 ± 5.9
9	5.15	0.75	150	29.4 ± 3.03	27.5 ± 4
10	5.5	0.5	106	26.1 ± 2.24	47.9 ± 4.5
11	5.15	0.75	150	30.7 ± 1.49	25.7 ± 2

**Table 3:** Comparison of the adsorption capacity of kikuyu and other biosorbents.

Adsorbent	q (mg/g)	Reference
*Algae spirogyra* spp.	30.21	[30]
Chitosan microfiber bayberry tannin	20.90	[31]
Arribadas algae	82.33	[32]
Native microalgae consortium	4.44	[21]
Yellow passion fruit shell	85.1	[27]
*Cymbopogon flexuosus*	121.64	[33]
*Zea mays* seed chaff	14.68	[34]
Rose waste biomass	67.34	[35]
Aerobic granules	37.8	[36]
Kikuyu	47.9	This work

**Table 4:** Estimated effects on adsorption capacity (q).

Effect	Estimated	Confidence Int.
Average	31.01	±1.42921
A: pH	3.86	±3.3518
B: Dose	−17.74	±3.3518
C: Size	−3.99	±3.3518
AB	−2.11	±3.3518
AC	−0.49	±3.3518
BC	1.77	±3.3518

AB denotes the pH–dose interaction, AC denotes the pH–size interaction, and BC denotes the dose–size interaction.

**Table 5:** Kinetic constant models.

Pseudo-First-Order Model		Pseudo-Second-Order Model		
R^2^	k_1_ (min^−1^)	R^2^	K_2_ (g∙mg^−1^∙min^−1^)	q_e_ (mg/g)
0.0852	0.0127	0.9995	0.194	41.49

**Table 6:** Variables and levels.

Independent Variables	Levels	
Size, *T* (μm)	106	212
Dose, *D* (mg/L)	0.5	1.0
pH	4.8	5.5

## Data Availability

The data are contained within the article.
